# Synbiotic-IgY Therapy Modulates the Mucosal Microbiome and Inflammatory Indices in Dogs with Chronic Inflammatory Enteropathy: A Randomized, Double-Blind, Placebo-Controlled Study

**DOI:** 10.3390/vetsci10010025

**Published:** 2022-12-30

**Authors:** Dipak Kumar Sahoo, Karin Allenspach, Jonathan P. Mochel, Valerie Parker, Adam Joseph Rudinsky, Jenessa A. Winston, Agnes Bourgois-Mochel, Mark Ackermann, Romy M. Heilmann, Gabor Köller, Lingnan Yuan, Tracey Stewart, Shannon Morgan, Kaitlyn R Scheunemann, Chelsea A. Iennarella-Servantez, Vojtech Gabriel, Christopher Zdyrski, Rachel Pilla, Jan S Suchodolski, Albert E. Jergens

**Affiliations:** 1 Department of Veterinary Clinical Sciences, College of Veterinary Medicine, Iowa State University, Ames, IA 50011, USA; 2 Department of Biomedical Sciences, College of Veterinary Medicine, Iowa State University, Ames, IA 50011, USA; 3 Department of Veterinary Clinical Sciences, College of Veterinary Medicine, The Ohio State University, Columbus, OH 43210, USA; 4 National Animal Disease Center USDA, ARS, Ames, IA 50010, USA; 5 Department for Small Animals, Veterinary Teaching Hospital, College of Veterinary Medicine, University of Leipzig, DE-04103 Leipzig, SN, Germany; 6 Department for Large Animals, University of Leipzig, DE-04103 Leipzig, SN, Germany; 7 Roy J. Carver High Resolution Microscopy Facility, Iowa State University, Ames, IA 50011, USA; 8 Gastrointestinal Laboratory, School of Veterinary Medicine and Biomedical Sciences, Texas A&M University, College Station, TX 77843, USA

**Keywords:** synbiotic, dog, chronic enteropathy, mucosal microbiota, fluorescence in situ hybridization, calprotectin

## Abstract

**Simple Summary:**

Chronic inflammatory enteropathy (CE) is a common cause for persistent or intermittent diarrhea and intestinal inflammation in dogs. Since there is evidence that imbalances in intestinal bacteria (dysbiosis) are associated with mucosal inflammation, probiotics, prebiotics, or a combination of the two (synbiotics) may reduce intestinal inflammation and decrease severity of dysbiosis. The goal of this study was to investigate the effects of dietary supplement synbiotic-IgY on clinical signs, inflammatory indices, and the mucosal microbiota in the dogs with CE. Twenty dogs with CE completed a prospective randomized clinical trial and were administered a hydrolyzed diet with the dietary supplement (synbiotic-IgY) or placebo (hydrolyzed diet) continuously for 6 weeks. At trial completion, results indicated that clinical disease activity and endoscopic scores decreased in both groups. Compared to dogs who were fed placebo, dogs administered supplement exhibited decreased levels of inflammatory markers fecal calprotectin and high-sensitivity C-reactive protein (hs-CRP) two weeks post-treatment, decreased levels of hs-CRP two- and six-weeks post-treatment, increased numbers of mucosal Clostridia and Bacteroides and decreased numbers of Enterobacteriaceae in colon biopsies at the completion of the trial. Results suggest that hydrolyzed diet and supplement effect favorable changes to host responses and the mucosal microbiota in dogs with CE.

**Abstract:**

Chronic inflammatory enteropathy (CE) is a common cause of persistent gastrointestinal signs and intestinal inflammation in dogs. Since evidence links dysbiosis to mucosal inflammation, probiotics, prebiotics, or their combination (synbiotics) may reduce intestinal inflammation and ameliorate dysbiosis in affected dogs. This study’s aim was to investigate the effects of the synbiotic-IgY supplement on clinical signs, inflammatory indices, and mucosal microbiota in dogs with CE. Dogs with CE were enrolled in a randomized prospective trial. Twenty-four client-owned dogs were fed a hydrolyzed diet and administered supplement or placebo (diet) for 6 weeks. Dogs were evaluated at diagnosis and 2- and 6-week post-treatment. Outcome measures included clinical activity, endoscopic and histologic scores, inflammatory markers (fecal calprotectin, C-reactive protein), and composition of the mucosal microbiota via FISH. Eleven supplement- and nine placebo-treated dogs completed the trial. After 6 weeks of therapy, clinical activity and endoscopic scores decreased in both groups. Compared to placebo-treated dogs, dogs administered supplement showed decreased calprotectin at 2-week post-treatment, decreased CRP at 2- and 6-week post-treatment increased mucosal Clostridia and Bacteroides and decreased Enterobacteriaceae in colonic biopsies at trial completion. Results suggest a beneficial effect of diet and supplements on host responses and mucosal microbiota in dogs with CE.

## 1. Introduction

Canine chronic inflammatory enteropathy (CE) denotes a group of intestinal disorders characterized by persistent or recurrent gastrointestinal (GI) signs and variable intestinal inflammation. Traditionally, CE is defined by the response to therapy with the most common classifications including food-responsive disease (FRD), antibiotic-responsive disease (ARD), and steroid-responsive disease (SRD), with this latter group often termed idiopathic inflammatory bowel disease (IBD) [1]. Affected dogs exhibit variable GI signs, including diarrhea, vomiting, alterations in appetite, and weight loss which may or may not be accompanied by excessive enteric plasma protein loss (i.e., protein-losing enteropathy). While the cause for canine CE is unknown, it is believed that it results from a complex interplay between the environment (e.g., diet, microbiome), mucosal immunity, and host genetics that initiate and drive chronic intestinal inflammation [2,3,4].

Recent evidence has emphasized the association between dysbiosis and intestinal inflammation in dogs with CE, and treatments attempting to reduce mucosal inflammation by normalizing dysbiotic states are a rapidly growing research area. Dietary trials using highly digestible, low-fat, and antigen-restricted or hydrolyzed protein diets ameliorate clinical signs in dogs with FRDt [5,6,7]; however, microbiome disruption remains after treatment [8]. Antibiotics are uncommonly required in dogs with CE since the incidence of ARD is low [9] and treatment relapses may occur once these drugs are discontinued [10]. Furthermore, antibiotic administration results in dysbiosis and contributes to the spread of antimicrobial resistance worldwide [11,12]. In dogs with SRD, steroid therapy with or without other treatments may effectively reduce GI signs; however, clinical recovery is not always accompanied by the correction of dysbiosis [13]. This information underscores the need for therapeutic options to improve GI dysbiosis, intestinal inflammation, and clinical signs in dogs with CE.

Probiotics, prebiotics, or their combination (synbiotics) and avian immunoglobulins are current focuses in GI research since they may reduce intestinal inflammation and aid in the recovery of dysbiosis in dogs with CE [14,15]. Probiotics contain live microorganisms that, when consumed in sufficient amounts, confer health benefits to the host [16]. Probiotics can alter the intestinal microbiota and provide beneficial effects on mucosal health in humans, rodents, and dogs with CE [17,18]. Prebiotics, often in the form of dietary fiber, are defined as a substrate selectively used by host microorganisms that confers a health benefit. Different compounds, including mannan-oligosaccharides, beta-glucans, D-mannose, and others have been shown to confer health benefits to the host and have a profound effect on the intestinal microbiome [19]. Immunoglobulin Y (IgY), the avian homolog of IgG in humans, has shown benefits in treating GI infectious diseases in humans and animals [20,21,22]. A commercially available dietary supplement containing a synbiotic combination with IgY (Intesto-Guard, IG Biosciences, Newton, IA 50208, USA) has recently been developed to correct GI dysbiosis, reduce GI signs, and target intestinal inflammation. We hypothesized that administration of the dietary supplement with hydrolyzed protein diet to dogs with CE would improve clinical disease activity, histopathology, endoscopic lesions, mucosal microbiota, and biomarkers of inflammation compared to placebo (hydrolyzed protein diet alone).

## 2. Materials and Methods

### 2.1. Ethical Considerations

Dogs with CE were enrolled in a multi-center (Iowa State University and Ohio State University) randomized, double-blind, placebo-controlled study from 2018–2021. As such, both clinicians and clients whose pet was being treated were blinded as to which treatment was administered. The animal use/clinical trial protocol was reviewed and approved by the IACUC committees of both institutions (ISU: IACUC-19-158; OSU: IACUC-2019A00000100). All clients gave written informed consent for their pet’s enrollment.

### 2.2. Animals

All dogs with CE were diagnosed based on stringent diagnostic criteria, excluding other causes for chronic GI signs [5,7]. These criteria included failed response to previous dietary and antimicrobial therapies for their GI signs, exclusion of metabolic/endocrine disorders, and biopsy confirmation of intestinal inflammation. Dogs could not have received glucocorticoids, antibiotics, or probiotics for at least 14 days prior to trial enrollment. Diagnostic tests to exclude other disorders included stool examination for endoparasites, complete blood count, biochemistry profile, urinalysis, serum folate, and cobalamin concentrations, serum trypsin-like immunoreactivity, serum pancreatic lipase immunoreactivity, resting cortisol and/or ACTH stimulation test, diagnostic imaging (abdominal ultrasound) and upper (gastroscopy, duodenoscopy) and lower (ileoscopy, colonoscopy) GI endoscopy for mucosal inspection and collection of biopsy specimens. Dogs were required to meet all inclusion criteria for CE diagnosis and exclusion of concurrent disease prior to enrollment in the clinical trial.

### 2.3. Synbiotic/IgY Supplement

Intesto-Guard™ (IG Biosciences, Inc., Newton, IA, USA) is a commercially available probiotic supplement designed for use in dogs, cats, and horses. It contains a blend of three products referred to as PPAs: Probiotics, Prebiotics, and Antibody IgY. The probiotics include *Lactobacillus acidophilus*‚ *Lactobacillus casei*‚ *Enterococcus faecium*, and *Bacillus subtilis* at a concentration of 1 billion CFU/mL (paste formulation). The prebiotics (yeast derivatives) include beta-glucans, mannan oligosaccharides (MOS), and D-mannose. The immunoglobulin IgY is derived from chicken egg yolk. The exact proportions of each component in the PPA formulation (bacterial strains, prebiotics, and IgY) are proprietary and the product is patented.

### 2.4. Study Design

**Figure 1:**
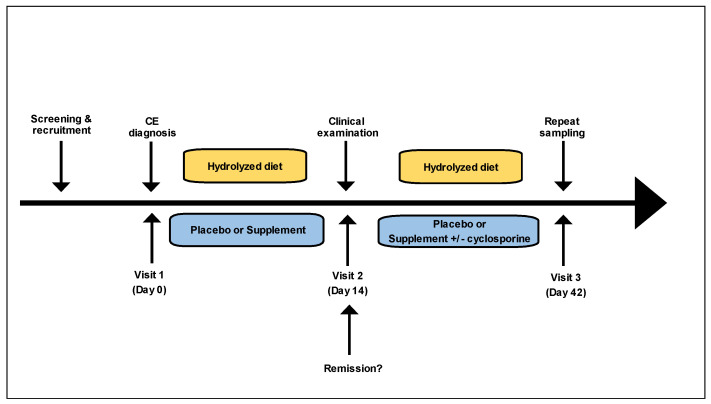
Trial design and clinical assessment. Laboratory and endoscopic analyses performed at visit 1 (day 0) and repeated at visit 3 (day 42). Assessment for remission and treatment escalation (with cyclosporine) was performed at visit 2 (day 14). Hydrolyzed diet was maintained in all dogs for the full treatment schedule of 42 days.

Dogs with confirmed CE were randomized by means of a computer-generated schedule into one of two treatment groups: synbiotic/IgY (supplement) and placebo. All dogs were transitioned to a hydrolyzed protein diet (Purina HA [first choice] or Royal Canin Hydrolyzed Protein Adult HP [second choice]) at enrollment and the diet was maintained throughout the study period (Figure 1). Dogs in the supplement group received a dose of 1 mL paste per 2.2 kg of body weight orally twice daily. Dogs in the placebo group received a placebo paste of identical color composed of excipients and flavorings dosed at 1 mL per 2.2 kg body weight orally twice daily. Dogs were evaluated at 3 timepoints: at diagnosis (pre-treatment, visit 1), after 2 weeks of treatment (post-treatment, visit 2), and after the treatment schedule was completed (6 weeks post-treatment, visit 3). Clinical remission was evaluated after 14 days of treatment (post-treatment, visit 2) and was defined as a 50% or greater reduction in the baseline clinical activity score [5]. At post-treatment visit 2, dogs failing remission maintained their original treatment but underwent treatment escalation with cyclosporine administered at 5 mg/kg PO q24h for one day, then increased to 5 mg/kg PO q12h for the remaining study period. Both owners and clinicians were blinded to all assigned treatments. The clinical trials specialist acted as the dispenser of all treatments.

Blood samples, urine, and feces were collected at baseline, after 14 days of treatment (post-treatment, visit 2), and at the conclusion of the treatment schedule (post-treatment, visit 3). Urine samples were processed immediately at each hospitalization visit. Gastrointestinal endoscopy with collection of mucosal biopsies was repeated on post-treatment, visit 3. Intestinal biopsies were placed in 10% neutral buffered formalin, routinely processed and paraffin embedded as a tissue block for H&E histopathologic assessment. Serum was collected by centrifugation, frozen within one hour, and stored at −80 °C until analysis. Fecal samples were collected at each time point and were stored at −80 °C for later microbiome and biomarker analyses.

### 2.5. Disease Activity Indices

Scoring for clinical disease activity was performed using the CCECAI score at diagnosis (pre-treatment, visit 1), after 2 weeks of treatment (post-treatment, visit 2), and after the treatment schedule was completed (post-treatment, visit 3) [5]. The simple endoscopic score was used for the assessment of mucosal lesions at diagnosis (pre-treatment, visit 1) and at the conclusion of the treatment schedule (post-treatment, visit 3) [23]. A single internist from each institution scored the endoscopic procedures at their institution. Endoscopic scores from each intestinal region (duodenum, ileum, and colon) were summed, yielding a total endoscopic score for each dog and endoscopic examination. Histopathologic examination of intestinal biopsies was performed by a single pathologist (MA) blinded to each dog’s clinical disease activity and treatment (supplement vs. placebo). Mucosal biopsies were assessed for intestinal inflammation using modified WSAVA histopathologic guidelines where morphologic and inflammatory features were graded and summed, yielding a total cumulative inflammatory score [24].

### 2.6. Mucosal Microbiota

Formalin-fixed embedded ileal and colonic tissue sections were mounted on glass slides and evaluated by fluorescence in situ hybridization (FISH) as previously described [25,26]. In brief, paraffin-embedded tissue specimens were deparaffinized using an automated system by passage through xylene (3 × 10 min), 100% alcohol (2 × 5 min), 95% ethanol (5 min), and finally 70% ethanol (5 min). The slides were transported in deionized water to the DNA testing laboratory, where they were air-dried before hybridization. FISH probes 5′ - labeled with either Cy-3 or FITC (Thermo Fisher Scientific, Rochester, USA) were reconstituted with nuclease-free water and diluted to a working concentration of 5 ng/mL. For total bacterial counts, EUB338-FITC was used [27]. For other analyses, specific probes directed against *Clostridium* (EREC482) [28], *Bacteroides* (BAC303) [29], and Enterobacteriaceae (EBAC1790) [30] were labeled with Cy-3 and applied simultaneously with the universal bacterial probe Eub338-FITC. The probe array was selected to identify specific bacterial groups previously shown to be relevant in the pathogenesis of chronic gastrointestinal inflammation in humans [18,31,32] and animals [33,34,35]. Tissue sections were immersed in 30 mL of DNA–probe mix in a hybridization chamber maintained at 54°C overnight (12 h). Washing was performed using a wash buffer (hybridization buffer without SDS); the slides were rinsed with sterile water, then allowed to air dry, and mounted with SlowFade Gold mounting media (Life Technologies, Carlsbad, CA, USA) and 25 × 25^−1^ cover glass (Fisher Scientific, Pittsburgh, PA, USA).

Probe specificity was confirmed in pilot studies by combining the irrelevant probe non-Eub338-FITC with Eub338-Cy-3-labeled probes, and through hybridization with pure bacterial cultures. Sections were examined on a Zeiss AxioImager Z.2 epifluorescence microscope (Dublin, CA, USA) and images were captured with a Zeiss MRM AxioCam camera (Dublin, CA, USA) (www.zeiss.com; accessed on 4 April 2022).

### 2.7. Biomarkers of Inflammation

Local (fecal calprotectin) and systemic (serum high sensitivity C-reactive protein [hs-CRP]) biomarkers of inflammation were evaluated in a subset of dogs at diagnosis (pre-treatment, visit 1, day 0) and after the treatment schedule was completed (post-treatment, visit 3, day 42). Fecal calprotectin was measured in samples from 12 dogs extracted to a final dilution of 1:500 (Calex Cap, Bühlmann Laboratories, Schönenbuch, BL, Switzerland) by a particle-enhanced turbidimetric immunoassay (fCAL turbo, Bühlmann) on a Cobas 311 chemistry analyzer (Roche Diagnostics GmbH, Mannheim, Germany) as described previously [36]. This assay has been validated for use with canine fecal samples and was shown to have a lower detection limit of 3 µg/g, a reference interval of 3−41 µg/g, and a minimum critical difference of 44.0% [36]. High-sensitivity C-reactive protein (hs-CRP) in serum was analyzed by sandwich ELISA according to the manufacturer’s instructions (MyBioSource Inc., San Diego, CA, USA). The concentration of hs-CRP in dogs with CE was compared to hs-CRP values obtained from a cohort (*n* = 20) of healthy dogs.

### 2.8. Statistical Analysis

Sample size calculation was performed prior to trial enrollment. It was estimated that 50% of dogs treated with diet alone would be in remission at 14 days. This hypothesis was based on results from previous studies that showed use of an elimination diet results in remission rates of 50–60% in dogs with CE [9,37]. A minimum clinically significant difference in the occurrence of this outcome between both treatment groups was estimated at 25%. Therefore, a remission rate of 75% in the supplement group was anticipated. Randomization of 15 dogs per group would give a power of 80% to detect this difference at the 0.05 significance level. Additional dogs were enrolled to allow for a non-compliance rate of up to 20%.

Statistical analysis was performed with the use of statistical software (R version 4.2.1). The Wilcoxon rank sum test was used for comparing numerical data (age, weight, gender) and histopathologic scores from visit 1 to visit 3. Clinical (CCECAI) and total endoscopic scores across the treatment schedules were analyzed using one-way ANOVA with Šídák’s multiple-comparisons test. Logistic regression was used to analyze the relationship between CCECAI, histology, endoscopy, duration of GI signs, and treatment escalation. Comparison of numbers of mucosal bacteria, fecal calprotectin, and hs-CRP values in response to treatment was performed with GraphPad Prism 9 (version 9.4.1) (https://graphpad.com/; accessed on 2 September 2022) using a two-tailed Student’s *t*-test. A *p*-value of <0.05 was considered significant for all analyses.

## 3. Results

### 3.1. Animals—Baseline Characteristics

**Figure 2:**
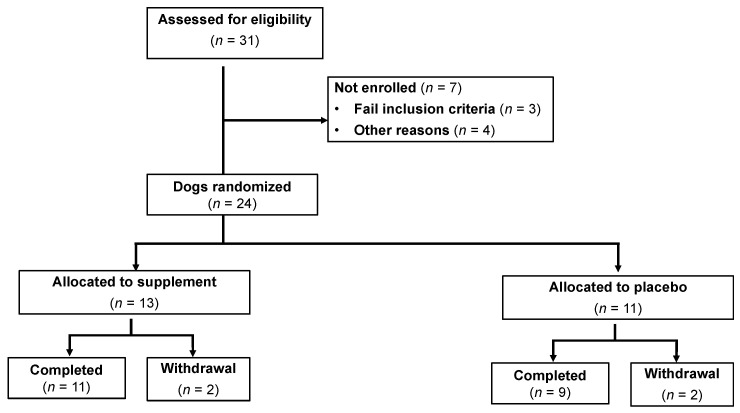
Dog enrollment and randomization for the clinical trial.

During the study period, 31 dogs were assessed for eligibility (25 from Iowa State University and 6 from Ohio State University), and 24 dogs with CE were enrolled in the trial (Figure 2). Four dogs failed to complete the trial due to portal vein thromboses (*n* = 2), too ill for repeat general anesthesia (*n* = 1), and one dog did not return for repeat GI endoscopy. The patient demographic data for both cohorts are shown in Table 1. Dogs with CE comprised the following breeds: Shih Tzu, Labrador Retriever (*n* = 2), Cardigan Welsh Corgi, Great Pyrenees, Pembroke Welsh Corgi, Vizsla, Yorkshire Terrier, Boston Terrier, German Shepherd dog, Norwegian Elkhound, Mongrel (*n* = 3), Cavalier King Charles Spaniel, Pitbull, Boxer, Great Dane, Bichon Frise, and Bernese Mountain dog. There were no significant differences (*p* > 0.05) in age, weight, gender distribution, or clinical disease activity between treatment groups at diagnosis (Table 2). Abnormal laboratory findings at diagnosis included decreased serum cobalamin (5 dogs), increased serum folate (4 dogs), and decreased serum folate (1 dog). Abnormal findings on abdominal ultrasound were observed in 6 dogs and included intestinal hyperechoic mucosal striations or speckles (3 dogs), scant abdominal effusion (3 dogs), increased thickness to the intestinal mucosa (5 dogs), and/or enlargement of mesenteric lymph nodes (3 dogs).

### 3.2. Clinical Scores

**Figure 3:**
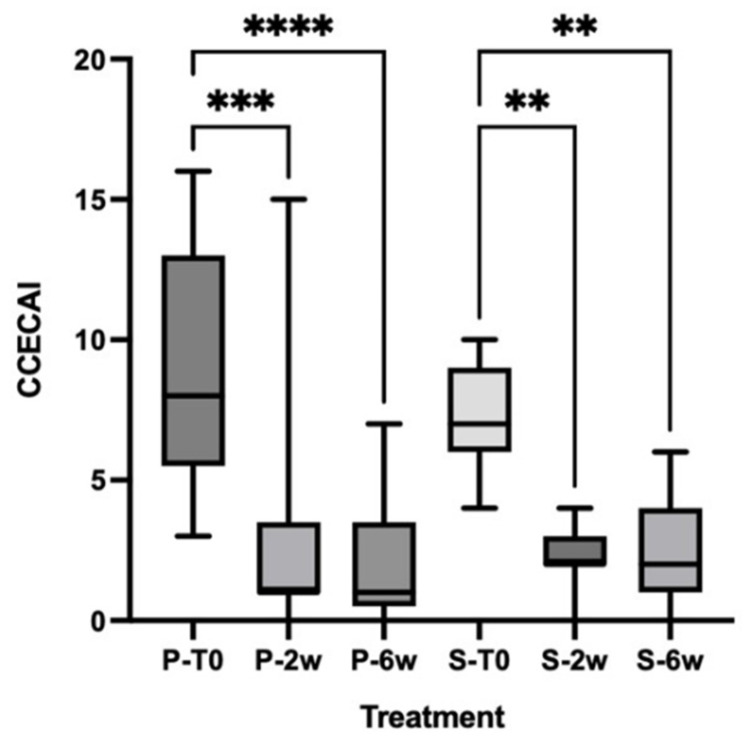
Clinical disease activity scores over the treatment schedule in placebo (P) and supplement (S) treated dogs with CE. The boxplots display the median and quartiles. T0 = pre-treatment values, 2w = 2 weeks post-treatment, 6w = 6 weeks post-treatment. P = placebo, S = supplement. **** significantly different at *p* value < 0.0001, *** significantly different at *p* value < 0.0002, ** significantly different at *p* value < 0.005.

There was no significant difference (*p* > 0.05) in clinical scores between dog groups at diagnosis. Most dogs had moderate-to-severe clinical disease activity. Collectively, both groups showed rapid and sustained remission during the study period (Figure 3). Compared with pre-treatment values; there was a significant decrease (*p* < 0.05) in clinical scores of both dog groups at the end of the treatment period. The median (and range) of clinical scores for supplement- and placebo-treated dogs after 2 weeks of treatment was 2 (0–4) and 1 (0–15), respectively. Four supplement-treated and one placebo-treated dogs failed to reach remission at the two-week evaluation necessitating treatment escalation with cyclosporine along with their original treatments. Regression analysis failed to identify any variables influencing early remission. There was no significant difference (*p* > 0.05) in clinical scores between groups at the end of the treatment period (supplement group: median = 2, range: 0–6; placebo group: median =1, range: 0–7).

### 3.3. Simple Endoscopic Score

**Figure 4:**
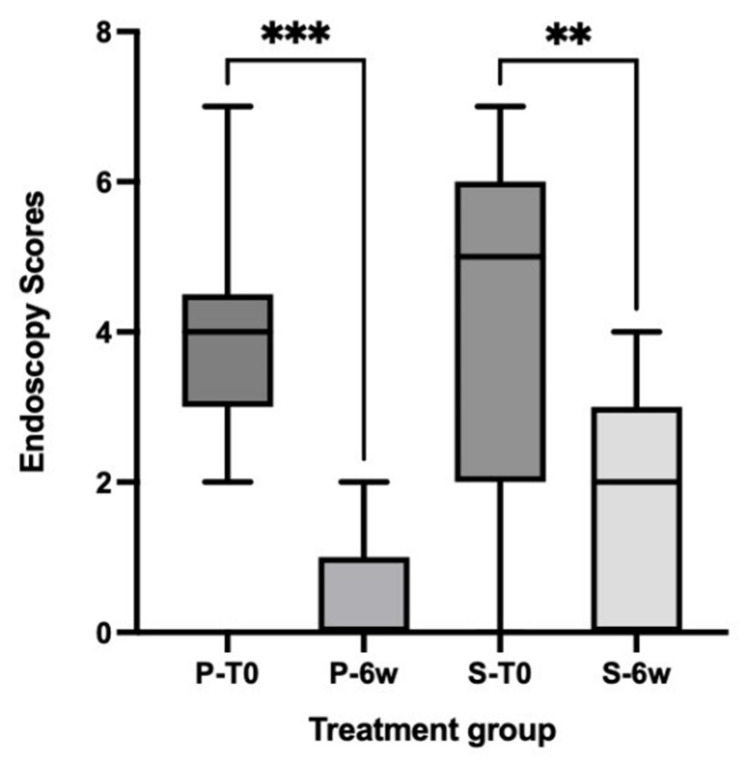
Total endoscopic scores over the treatment schedule in placebo (P) and supplement (S) treated dogs with CE. The boxplots display the median and quartiles. T0 = pre-treatment, 6w = 6 weeks post-treatment. *** significantly different at *p* value < 0.0002, ** significantly different at *p* value < 0.005.

There was no significant difference (*p* > 0.05) in endoscopic scores between treatment groups at diagnosis and after treatment. Abnormalities to the intestinal mucosa (i.e., erosions, friability, granularity, white speckles/spots) indicative of active intestinal inflammation and/or lymphangiectasia were commonly observed in both dog groups. Compared with pretreatment scores, both groups showed significantly decreased (*p* < 0.05) endoscopic scores accompanied by a marked reduction in mucosal abnormalities (indicative of mucosal healing) at the completion of the treatment period (Figure 4).

### 3.4. Histologic Findings

Using modified WSAVA histopathologic guidelines, dogs with CE were diagnosed with mild-to-moderate intestinal inflammation. Lymphoplasmacytic inflammation was the predominant inflammatory feature, with intestinal crypt abnormalities (i.e., abscess, dilation, hypertrophy) being a frequently observed morphologic lesion along with lymphatic dilation. There was no difference (*p* > 0.05) in total histologic scores pre-treatment between dog groups (supplement group: median = 2, range: 1–3; placebo group: median =1, range: 1–3). Compared with pre-treatment total histology scores, four of nine placebo dogs and seven of eleven supplement dogs had decreased total histology scores post-treatment. When groups were compared at the end of the treatment period, there was no significant difference (*p* > 0.05) in post-treatment total histologic scores (supplement group: median = 1, range: 1–2; placebo group: median =1, range: 1–2).

### 3.5. Mucosal Microbiota

**Figure 5:**
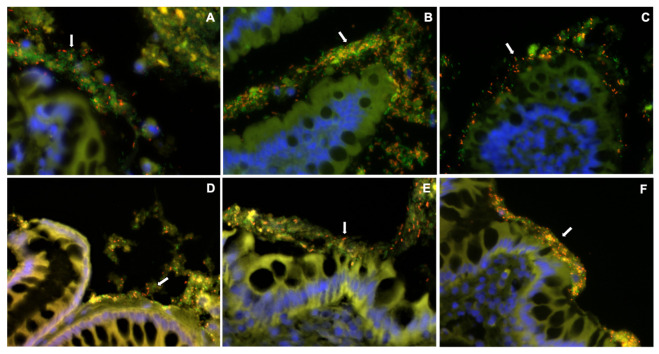
Three-color FISH identifies adherent mucosal bacteria present in canine ileum (**A**–**C**) and colon (**D**–**F**) endoscopic biopsies. Specific bacterial groups (Bacteroides [BAC303], Clostridia [EREC482], and Enterobacteriaceae [EBAC1790]) hybridizing with Cy3 appear orange. All other bacteria hybridizing with the universal probe (EUB-FITC) appear green. DAPI-stained colonic mucosa with nuclei stain blue. **Panel A**—Post-treatment, Supplement, EBAC; **Panel B**—Pre-treatment, Placebo, BAC; **Panel C**—Post-treatment, Placebo, BAC; **Panel D**—Post-treatment, Supplement, BAC; **Panel E**—Pre-treatment, Placebo, EBAC; **Panel F**—Pre-treatment, Placebo, BAC. Figure arrows indicate numerous bacterial colonies residing within adherent mucus. All images at 60× magnification.

**Figure 6:**
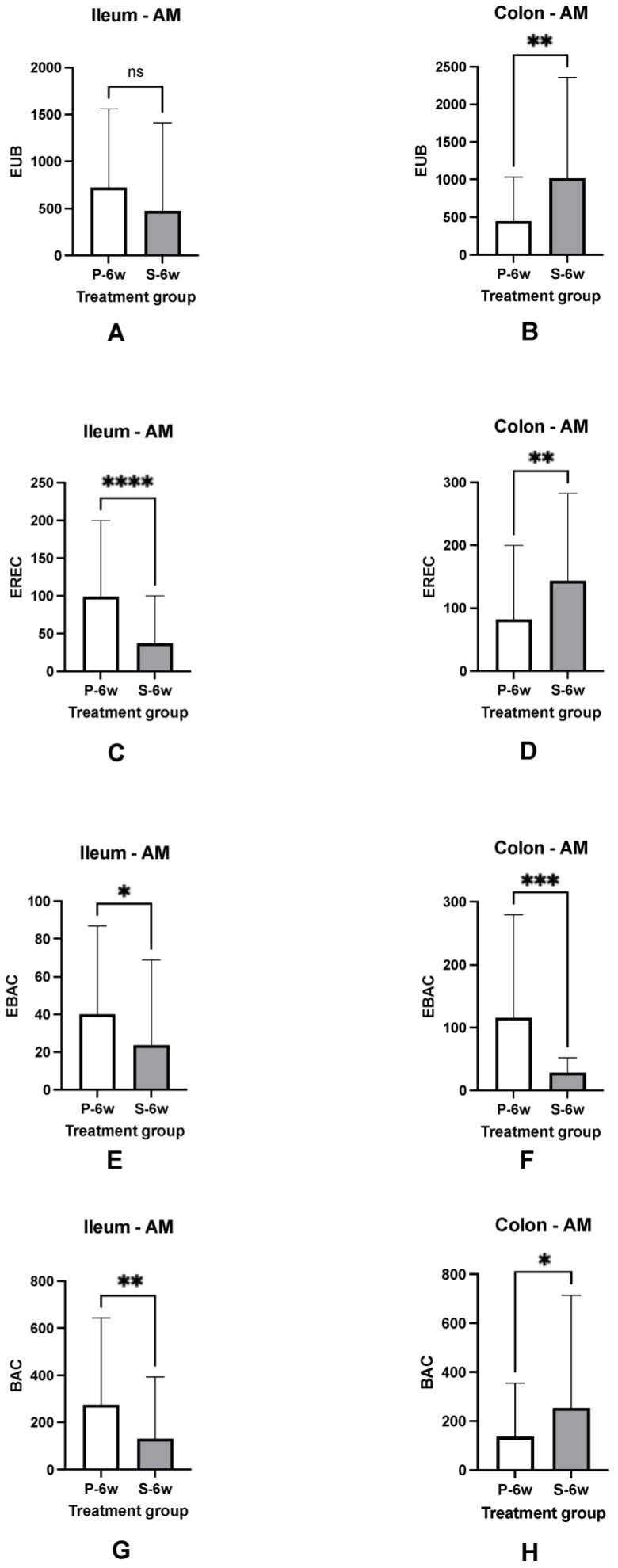
Numbers of mucosal bacteria in ileal and colonic biopsies of dogs with CE at trial completion (6 weeks post-treatment [6w]). Data expressed as mean ± standard deviation. EUB = total bacteria (**Panel A**,**B**), EREC = Clostridia (**Panel C**,**D**), EBAC = Enterobacteriaceae (**Panel E**,**F**), BAC = Bacteroides (**Panel G**,**H**). P = placebo, S = supplement. **** significantly different at *p* value < 0.0001, ** significantly different at *p* value < 0.01, * significantly different at *p* value < 0.05. ns = no significant difference. AM = adherent mucus.

The mucosal microbiota of the ileum and colon was most abundant in the adherent mucus of both dog groups at diagnosis. Sub-populations of bacteria hybridized with probes targeting *Clostridium* spp., *Bacteroides* spp., and Enterobacteriaceae. There was no significant difference (*p* > 0.05) in the total number of EUB338-positive bacteria between dog groups or intestinal regions at diagnosis. Following treatment, ileal biopsies of placebo-treated dogs contained increased (*p* < 0.05) numbers of *Clostridium* spp. and Enterobacteriaceae compared to ileal biopsies from supplement-treated dogs. Dogs with CE that received synbiotic therapy also had much higher numbers of *Clostridium* spp. and *Bacteroides* spp., (*p* < 0.05), localized predominantly within adherent mucus of colonic biopsies, than placebo-treated dogs. The numbers of Enterobacteriaceae were less numerous (*p* < 0.05) within colonic biopsies of dogs receiving synbiotic therapy as compared to placebo-treated dogs (Figure 5 and Figure 6).

### 3.6. Biomarkers of Inflammation

**Figure 7:**
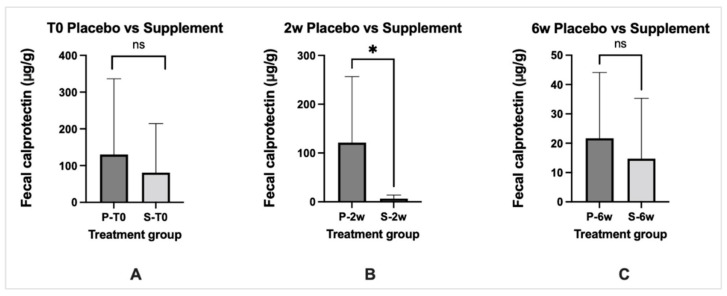
Fecal calprotectin levels over the treatment schedule in placebo (P) and supplement (S) treated dogs with CE. Data expressed as mean ± standard deviation. Panel A = T0 (pre-treatment) values, Panel B = 2 weeks (2w) post-treatment, Panel C = 6 weeks (6w) post-treatment. * significantly different at *p* value < 0.05. ns = no significant difference. Fecal calprotectin reference interval: 3–49 ug/g.

Fecal calprotectin levels in both dog groups were increased above the reference interval pre-treatment but were not significantly different (*p* > 0.05). There was a significant (*p* < 0.05) decrease in fecal calprotectin levels in the supplement-treated dogs at 2-week post-treatment when compared to placebo-treated dogs. When compared with pre-treatment levels, fecal calprotectin levels decreased significantly (*p* < 0.05) to the reference interval in both groups post-treatment but were not significantly different (*p* > 0.05) between dog groups (Figure 7).

**Figure 8:**
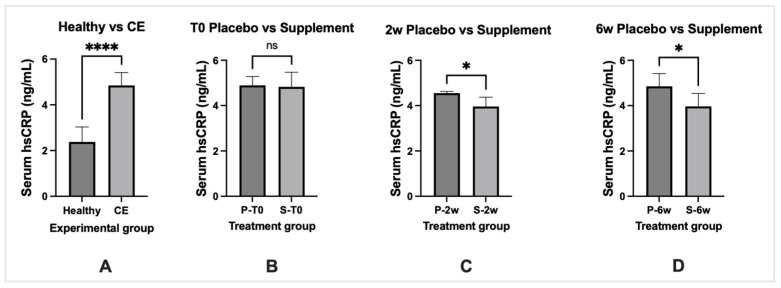
Serum high sensitivity CRP over the treatment schedule in placebo (P) and supplement (S) treated dogs with CE. Healthy dogs (*n* = 20) comprised a cohort of young adult dogs free of GI signs. Data expressed as mean ± standard deviation. Panel A = comparison of healthy dogs vs. dogs with CE, Panel B = T0 (pre-treatment) values, Panel C = 2 weeks (2w) post-treatment, Panel D = 6 weeks (6w) post-treatment. **** significantly different at *p* value < 0.0001, * significantly different at *p*-value < 0.05. ns = no significant difference.

Dogs with CE had significantly elevated (*p* < 0.05) serum hs-CRP levels compared to serum hs-CRP levels of healthy dogs. Healthy dogs comprised a cohort of 20 young (1–4 years of age) adult dogs seen by the ISU Primary Care Service for recommended vaccinations. All healthy dogs had normal physical examinations and were disease-free at the appointment. Serum levels of hs-CRP were increased but not significantly different (*p* > 0.05) between treatment groups at diagnosis. Compared with pre-treatment levels, supplement-treated dogs showed significantly decreased (*p* < 0.05) hs-CRP levels at 2- and 6-week post-treatment as compared to placebo-treated dogs (Figure 8).

## 4. Discussion

There has been considerable interest in the clinical and therapeutic implications of intestinal dysbiosis in chronic GI diseases, including dogs with CE. Microbial imbalances may be a cause or consequence of chronic intestinal inflammation, with most dogs sharing a similar pattern of dysbiosis when compared to healthy dogs. More specifically, both luminal and mucosal intestinal bacteria have shown a common dysbiotic profile characterized by decreased abundance of *Fusobacterium*, *Clostridium*, and *Bacteroides*, and an increased abundance of Enterobacteriaceae [26,33,34,35,38,39]. Dogs with CE also exhibit significantly decreased fecal bacterial richness and diversity across different studies [40,41]. To aid in the correction of microbial perturbations in dogs with CE, treatment with pre- and probiotics or their combination (synbiotics) offers an attractive, non-pharmacologic option to decrease intestinal inflammation and promote clinical remission.

The objective of the current study was to investigate the effects of 6 weeks of administration of the synbiotic/IgY (supplement) together with a hydrolyzed protein diet on clinical disease activity, histopathology, endoscopic lesions, the mucosal microbiota, and biomarkers of inflammation in dogs with CE compared to the effects of hydrolyzed diet (placebo) alone. Both supplement and placebo treatment resulted in the remission of disease activity and endoscopic lesions at the completion of the treatment period. Moreover, total histologic scores for intestinal inflammation were not significantly different pre- versus post-treatment between dog cohorts. Compared to baseline values, the inflammatory biomarker fecal calprotectin was decreased 2 weeks post-treatment, and hs-CRP concentrations were decreased throughout the treatment period in dogs with CE receiving the supplement. While alterations in mucosal microbiota were observed in both groups after treatment, colonic biopsies of dogs treated with supplements showed beneficial changes in the number and composition of the mucosa-associated microbiota as compared to colonic biopsies from placebo-treated dogs. Collectively, these preliminary findings suggest that the dietary supplement is associated with favorable effects on host responses and the intestinal mucosal microbiota in dogs with CE.

Synbiotics offer an innovative strategy for reducing mucosal inflammation and correcting microbial imbalances in dogs with CE. A mixture of probiotics and prebiotics may beneficially impact the host through improved survival and colonization of live microbial organisms in the gut or by stimulating the growth and/or metabolism of one or more health-promoting bacteria [42]. There are limited studies evaluating treatment with probiotics or synbiotics in dogs with CE. Studies have demonstrated the benefit of high-potency multi-strain probiotics to mucosal health when administered alone [43] or in combination with standard therapy (i.e., elimination diet and prednisone) in dogs with IBD [25]. A synbiotic product composed of one strain of *Enterococcus faecium* (EF), fructooligosaccharides, and gum Arabic has been investigated in dogs with FRD in separate studies. The combination of this synbiotic combined with a hydrolyzed protein diet failed to affect cytokine protein production in ex vivo stimulated duodenal biopsies [44]; had no effect on clinical efficacy, histology scores, or immunologic gene expression [45]; and did not significantly alter fecal microbiota richness or diversity in dogs when administered over 6 weeks [46]. Finally, the yeast probiotic *Saccharomyces boulardii* (as part of a synbiotic preparation) was shown to improve clinical severity and stool frequency at days 45 and 60 when administered to IBD dogs with and without protein-losing enteropathy (PLE) [47]. In the PLE subgroup, 3/3 of dogs treated with *S. boulardii* showed improvement in serum albumin versus 2/3 of dogs in the placebo group.

A combination of clinical, endoscopic, and laboratory indices was used to define intestinal inflammation in dogs with CE at diagnosis and following treatment. Both supplement and placebo treatments, along with hydrolyzed protein diet, were associated with rapidly improved clinical scores, like previous studies [25,43]. Most dogs were in remission at 14 days post-treatment with no difference in disease severity at the completion of the treatment schedule. The simple endoscopic score was used to assess mucosal healing in dogs with intestinal inflammation, which is an important treatment endpoint in human IBD [48,49]. Both dog groups showed moderate-to-severe mucosal inflammation at diagnosis that was reduced following either treatment. There are few studies evaluating severity of mucosal inflammation in dogs with CE. In one prospective study investigating dogs with FRD, SRD, and non-responders requiring treatment escalation with cyclosporine, severe duodenal inflammation (e.g., friability, erosions, granularity) was associated with negative long-term outcome^5^. A similar study investigated the clinical, endoscopic, and histologic response to treatment in non-hypoproteinemic dogs with lymphoplasmacytic enteritis and showed improved gastric and duodenal lesions indicative of mucosal healing after medical therapy [50]. Accordingly, these studies suggest that GI endoscopy is useful in assessing mucosal healing and remission in dogs with CE.

Canine fecal calprotectin (CP) and hs-CRP were investigated as local and systemic biomarkers, respectively, of intestinal inflammation. They were chosen due to their utility as inflammation biomarkers previously in dogs with CE [51,52,53] and in human IBD [54,55]. Serum CRP is a positive acute-phase protein produced by the liver following stimulation by IL-6 and IL-1β in response to infection, inflammation, or cancer [56]. It has been investigated previously as a biomarker of disease severity and response to treatment in dogs with SRD [52,53]. Calprotectin is a mucosal-derived inflammatory protein shown to be associated with acute and chronic inflammation in dogs [53,57,58]. In the present study, CP was increased in dogs with CE at diagnosis but decreased to reference interval regardless of treatment. We also observed that serum hs-CRP, increased in dogs with CE at diagnosis, significantly decreased at 2- and 6-week post-treatment in dogs receiving the supplement. Our findings are in broad agreement with other studies showing that the combination of fecal CP and serum CRP are useful surrogate markers of disease severity and treatment response in dogs with CE [51,53].

This study is the first report describing the effects of synbiotic-IgY supplement as an adjunct treatment for dogs with CE. Immunoglobulin Y is the main antibody found in birds, amphibians, and reptiles and is composed of two heavy and light chains with constant and variable regions [22]. It is derived from the chicken egg yolk with an immunized hen laying approximately 300 eggs and producing 18–25 g of IgY per year [59]. IgY constitutes a relevant antibody source for clinical applications in veterinary medicine, which can be produced quickly, safely, and at a relatively low cost [60].

Polyclonal IgY has been previously used to treat different GI infectious diseases in humans and animals. While enteropathogens are not routinely associated with canine CE (excluding granulomatous colitis in Boxer dogs [39]), IgY may serve as a potential therapeutic option in enteropathogenic settings. In animal studies, specific IgY directed against *Salmonella typhimurium* reduced mucosal expression of proinflammatory cytokines TNF-α and IFN-γand elevated anti-inflammatory cytokine IL-10 in *S. typhimurium*-infected mice [61]. Moreover, mice treated with anti-*S. typhimurium*-IgY had reduced mucosal inflammatory infiltrate and prolonged survival versus mice treated with non-specific IgY. In another study, IgY directed against *Clostridiodes difficile* spores reduced the onset of diarrhea in rats prior to infection and reduced disease recurrence in infected rats [62]. The efficacy of IgY for the treatment of *Helicobacter pylori* infection has also been studied in animal models and humans. Here, IgY given against *H. pylori* urease (HPU) protein reduced *H. pylori* activity in infected Mongolian Gerbils and prevented colonization of the bacterium in the GI tract of controls [63]. Interestingly, an egg yolk powder supplement containing anti-HPU IgY reduced levels of *H. pylori* and the severity of gastritis in a cohort of asymptomatic *H. pylori*-positive human patients [64]. Still, other uses for IgY demonstrated in animal models include the treatment of dental caries [65], skin-related infections [66], and parasitic diseases [67].

Abundant evidence demonstrates that imbalances in intestinal microbiota are common in dogs with CE. However, most of these studies have analyzed the fecal microbiota [38,40,41,68] with relatively few investigations analyzing the mucosal microbiota [25,26,39]. In the present study, dogs treated with a supplement containing four different strains of probiotic bacteria, prebiotics, and IgY, showed significant changes in their mucosal microbiota compared to placebo-treated dogs. Supplement-treated dogs had increased numbers of colonic mucosal *Clostridium* spp. and *Bacteroides* spp. but decreased numbers of mucosal Enterobacteriaceae when compared to dogs administered placebo. Our data confirm previous observations in mucosal biopsies of humans with IBD [32,69] and dogs with CE [25,26,34,35], where a significant reduction in the numbers of *Clostridium* spp. and *Bacteroides* spp. are present at diagnosis. Recent studies suggest a beneficial role for these bacterial phyla as key producers of short-chain fatty acids (SCFA) [70]. Previous studies have shown that SCFA, such as butyrate, are energy sources for colonocytes and play an important role in maintaining intestinal epithelial barrier integrity [32]. In addition, overrepresentation of mucosal Enterobacteriaceae was observed in dogs with CE pre-treatment but was significantly reduced in colonic biopsies of dogs receiving the supplement. Enterobacteriaceae are considered harmful species due to their ability to trigger innate immune responses in the gut [71]. Our microbiologic results are different than a previous report comparing the effect of standard therapy (elimination diet and prednisone) with and without probiotics on the mucosal microbiota of dogs with CE25. In this previous study, there was no difference in the number of adherent mucus bacteria, rate of remission, and histopathologic inflammation between treatment groups. However, IBD dogs receiving probiotics did demonstrate increased expression of intestinal tight junction proteins suggesting a beneficial effect of probiotics on mucosal homeostasis.

There are some potential limitations to this study. The clinical trial lacked sufficient power to definitively define the effect of treatment between dog groups. A sample size calculation was performed with a biostatistician [52] (indicating enrollment of 30 total dogs with additional dogs to compensate for “washout”) and was based on initial power calculations using variability in treatment response to a hydrolyzed protein diet (placebo) alone [5,6,7]. While the number of dogs initially enrolled did meet this minimum requirement, the number of dogs completing the trial failed to reach statistical power for definitive conclusions. Nevertheless, it is likely that our results regarding the effect of treatment on mucosal bacterial populations are correct. The bacterial groups chosen for FISH analysis may not have included key phyla or new species associated with chronic intestinal inflammation [72]. However, previous studies in dogs with chronic GI signs have consistently shown patterns of mucosal [25,26,39] and luminal [38,40,41,68] dysbiosis involving Clostridia, Bacteroides, and Proteobacteria (Enterobacteriaceae), suggesting that our probe selection was appropriate. The precise role that hydrolyzed protein diet played in remission was not investigated in this trial. Therapeutic (hydrolyzed protein) diets can favorably modify the composition and/or function of the microbiome implicated in canine CE pathogenesis and host inflammation [73]. This could explain, at least partially, the similar responses in clinical and endoscopic scores observed between treatment groups. While both cohorts in the present study were fed a hydrolyzed protein diet with some dogs also receiving cyclosporine, only dogs administered the synbiotic showed beneficial changes in their mucosal microbiota.

In conclusion, our results suggest a beneficial effect of the supplement on host responses in dogs with CE as evidenced by decreased fecal CP, hs-CRP, and favorable changes in the mucosal microbiota. However, the effect of the supplement on clinical activity, mucosal healing, and histologic inflammation was like placebo. The dietary supplement containing synbiotic-IgY administered with diet was safe and well tolerated in dogs with CE. The present study provides preliminary data in a small patient cohort and should be replicated using larger randomized clinical trials to confirm these results.

## Figures and Tables

**Table 1:** Summary statistics for enrolled dogs with CE.

Dog.	Breed	Age (years)	Weight (kg.)	Sex	Treatment
1	Shih Tzu	10.9	5.9	FS	Supplement
2	Labrador Retriever	8.9	41.7	MC	Placebo
3	Cardigan Welsh Corgi	9.1	14.5	F	Placebo
4	Great Pyrenees	2.2	29.0	MC	Supplement
5	Pembroke Welsh Corgi	12.4	10.2	FS	Placebo
6	Labrador Retriever	5.9	33.0	M	Supplement
7	Vizsla	3.9	18.4	FS	Supplement
8	Yorkshire Terrier	5.8	6.8	MC	Supplement
9	Boston Terrier	13.0	8.6	FS	Supplement
10	Mongrel	1.1	19.4	MC	Supplement
11	German Shepherd Dog	4.7	51.0	MC	Supplement
12	Norwegian Elkhound	4.2	25.2	M	Placebo
13	Cavalier King Charles	11.7	7.3	FS	Placebo
14	Pitbull	7.3	21.4	MC	Placebo
15	Bichon Frise	5.1	5.3	MC	Supplement
16	Great Dane	5.0	38.0	MC	Placebo
17	Mongrel	1.1	36.0	FS	Supplement
18	Mongrel	2.3	46.1	MC	Supplement
19	Boxer	4.1	21.1	MC	Placebo
20	Bernese Mountain Dog	4.0	21.4	FS	Placebo

FS = female spayed; MC = male castrated; F = female; M = male.

**Table 2:** Baseline parameters of dogs with CE completing the treatment trial.

Parameter	Placebo Group (*n* = 9)	Supplement Group (*n* = 11)	Wilcoxon Statistic
Median age (years) Range	7.3 (4.1–12.4)	4.7 (1–12.9)	*p* > 0.05
Median Weight (kg.) Range	21.4 (7.3–41.7)	19.4 (5.3–51)	*p* > 0.05
Male sex, *n* (%)	5 (56)	7 (64)	*p* > 0.05
Median disease duration (mo.) Range	11 (2–36)	6 (1–42)	*p* > 0.05
Median CCECAI Range	8 (3–16)	7 (4–10)	*p* > 0.05
Number of PLE dogs Median serum albumin Range	1 1.4 g/dL * (1.3–2.3 g/dL)	3 1.5 g/dL (1.3–2.6 g/dL)	*p* > 0.05

* Median value assigned from 3 separate visits during the treatment period.

## Data Availability

The data presented in this study are available in the table and figure.
